# Optimal allocation of distributed energy storage systems to enhance voltage stability and minimize total cost

**DOI:** 10.1371/journal.pone.0296988

**Published:** 2024-01-29

**Authors:** Ramy Mohamed Hany, Tarek Mahmoud, El Said Abd El Aziz Osman, Abo El Fotouh Abd El Rehim, Hatem M. Seoudy

**Affiliations:** 1 Department of Electrical Power and Machines Engineering, The Higher Institute of Engineering, Elshorouk City, Egypt; 2 Department of Electrical Engineering, Al- Azhar University, Cairo, Egypt; National Institute of Technology Silchar, India, INDIA

## Abstract

The enhancement of energy efficiency in a distribution network can be attained through the adding of energy storage systems (ESSs). The strategic placement and appropriate sizing of these systems have the potential to significantly enhance the overall performance of the network. An appropriately dimensioned and strategically located energy storage system has the potential to effectively address peak energy demand, optimize the addition of renewable and distributed energy sources, assist in managing the power quality and reduce the expenses associated with expanding distribution networks. This study proposes an efficient approach utilizing the Dandelion Optimizer (DO) to find the optimal placement and sizing of ESSs in a distribution network. The goal is to reduce the overall annual cost of the system, which includes expenses related to power losses, voltage deviation, and peak load damand. The methods outlined in this study is implemented on the IEEE 33 bus distribution system. The outcomes obtained from the proposed DO are contrasted with those of the original system so as to illustrate the impact of ESSs location on both the overall cost and voltage profile. Furthermore, a comparison is made between the outcomes of the Ant Lion Optimizer (ALO) and the intended Design of Experiment DO, revealing that the DO has obtained greater savings in comparison to the ALO. The recommended methodology’s simplicity and efficacy in resolving the researched optimization issue make the acquired locations and sizes of ESSs favorable for implementation within the system.

## 1. Introduction

Significant changes are being forced upon the present distribution networks by a number of related factors, including demand management, integration of renewable energy, power quality standards, targets for reducing greenhouse gas emissions, network growth, and reliability [1–12]. According to the U.S. Electric Power Research Institute (EPRI), outages caused by problems with the distribution system are expected to cost approximately 100 billion USD annually [13]. The issues caused by power oscillations, disruptions to the distribution and transmission networks as well as sudden changes in load are anticipated to be successfully resolved by ESSs [14–16]. ESSs are being inserted in distribution networks to achieve Improvements in power quality, network expansion, cost savings, operating reserves, and a decrease in greenhouse gas emissions.

Additional benefits of ESSs include peak shaving, load shifting, load levelling, and voltage deviation mitigation [17–24]. Even while producing electricity from renewable energy is more ecologically beneficial, a strong reliance on it might impair the reliability of power distribution networks. With the help of energy-storage systems (ESSs), this issue with the integration of renewable energy sources may be resolved by reducing output variations, coordinating supply and demand, and balancing network power flow. Consequently, there is great potential for ESS implementation from the utility and consumer perspectives. In distribution networks, incorrect ESS location or sizing can have an impact on load management, frequency and voltage regulation, power quality, and reliability [25–37].

In a previous study [38], a multi-objective optimization problem was introduced, which integrates economic and technological objectives, with the aim of identifying the most optimal ESSs for distribution networks. The authors have presented a multi-objective function that incorporates network losses, bus voltage and energy cost in their work [39, 40]. The utilization of a particle swarm optimization (PSO) algorithm has been documented in previous studies [41, 42]. Previous research have shown the application of a genetic algorithm (GA) [43, 44]. The goal function, which incorporates the whole annual cost, was optimized using a Grey Wolf Optimizer (GWO) in the study done by [45]. The cost in question encompasses three components: the cost of energy not supplied, the cost of investing in ESSs, and the cost of operating the ESSs.

The suggested Dandelion Optimizer (DO)-based approach for optimal ESS location and scaling in the distribution network is demonstrated in this research. DO was chosen because to the necessity for easy implementation, quick reaction, and fewer controlling factors. A restricted objective function of the yearly cost (minimum) that incorporates the power loss cost, voltage variation cost, and peak demand cost has been constructed. The proposed approach is applied to the 33-bus system. A comparison is made between the findings acquired with the DO and those obtained with the ALO [46]. The proposed technique results in a lower total cost.

### 2. Ess modelling in distribution networks

**Fig 1:**
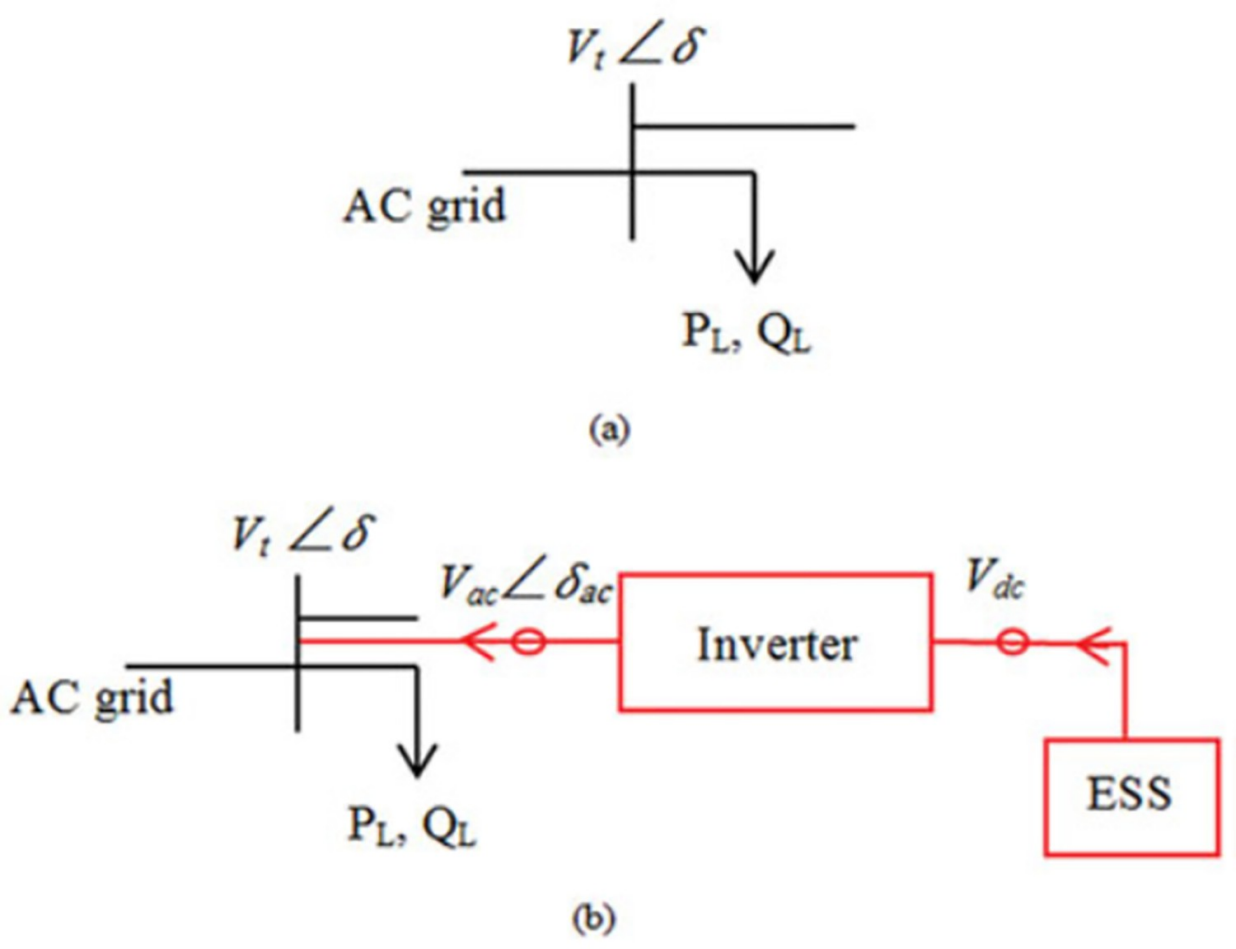
Depicts a distribution network segment (a) without and (b) with ESS.

In order to make up for the energy deficit that occurs when the electric networks operate outside of normal parameters, ESSs are technological devices designed to store electrical energy. As previously stated, the increased focus on ESSs may be attributed to their role in enhancing network dependability, augmenting network load capacity, and mitigating the intermittency of renewable energy sources. Fig 1(A) shows a standard bus connected to AC grid with Vt as terminal voltage and δ as angle supplies a load defined with active and reactive power *P*_*L*_ and *Q*_*L*_, while Fig 1(B) shows the standard bus with the use of an inverter is employed to establish a connection between the ESSs and the bus. This is achieved by converting the DC output voltage of the battery into AC voltage.

The transmission of both reactive and active power between the inverter terminal and the bus may be managed by regulating the AC voltage that the inverter derives. Consequently, the bus voltage and inverter terminal voltage magnitudes and angles determine how much active and reactive energy an ESS may create or consume. Considering the analysis presented above, it is possible to conceptualize ESSs as either generators or regulated loads due to their ability to store energy during the charging process and subsequently release it during discharge. The load level has an impact on the charging/discharging process. The state of ESSs is contingent upon the daily energy consumption. Consequently, during periods of insufficient energy supply, particularly when the demand is at its peak, ESSs operate as generators, redistributing the load among themselves. On the other hand, ESSs are presently in the process of being charged to store energy for excess power, particularly during periods of low demand. In the realm of load management, it is observed that ESSs are efficiently disengaged from the power grid when the load levels are within the specified range including the maximum and minimum load levels.

**Fig 2:**
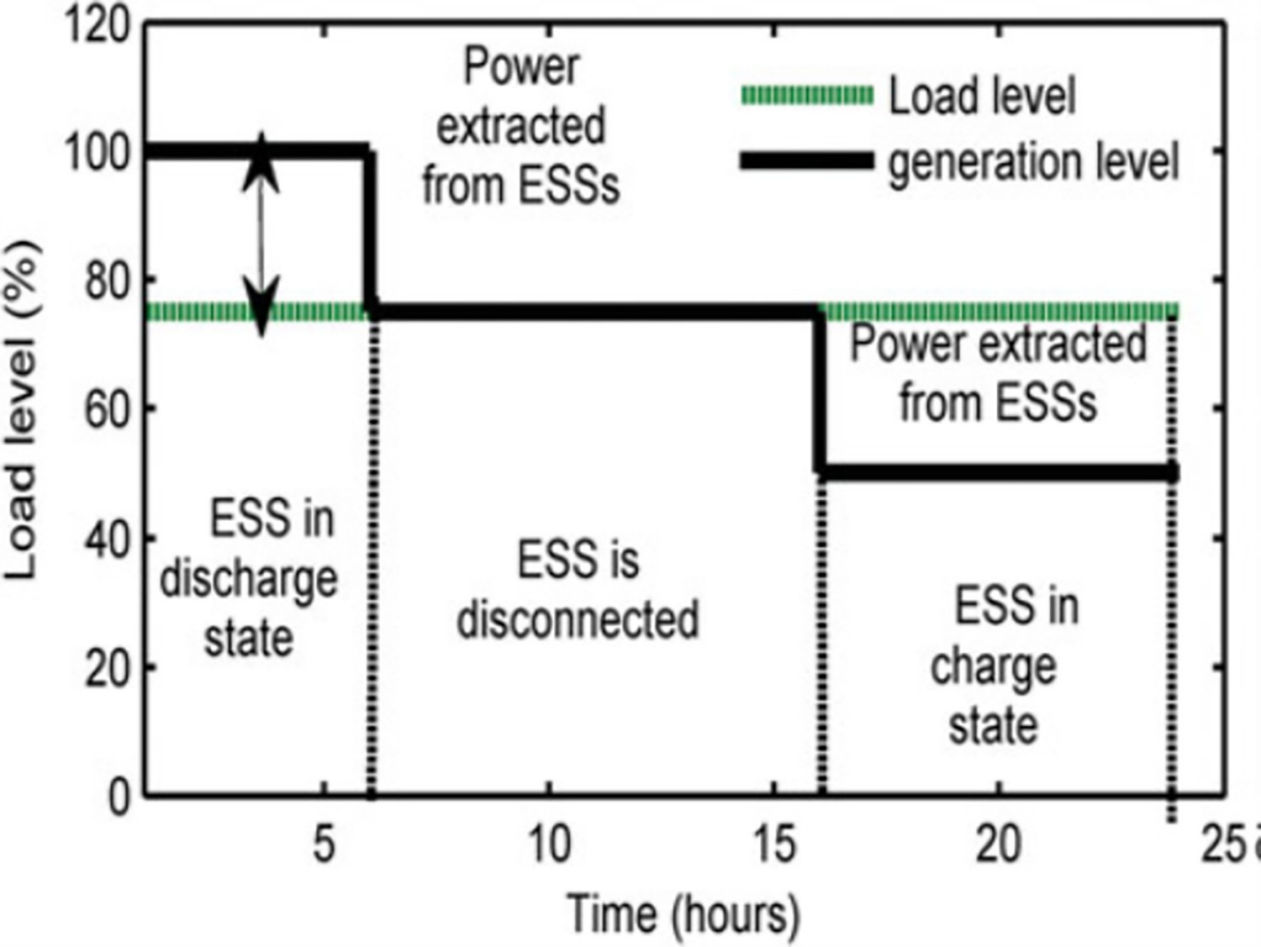
Typical ESS performance dependent on load level based on [42].

Based on the typical ESS performance provided in [42], Fig 2 shows the lowest and maximum load levels, which were chosen to be 50% and 75%, respectively. The hypothesis states that the network’s producing units can only supply 75% of the reference load, or the total installed load for a given day. Positive active power is detected during the discharging phase and negative active power is noticed during the charging phase of the (ESS), which is represented in the system as a PQ bus. Reactive power may show a positive or negative number in each of the provided scenarios.

The charging and discharging rates of ESSs are calculated as [47]:

$$\Delta E_{B}=E_{B}(t)-E_{B}(t-1)$$ (1)

$$P_{ch}(t)=\Delta E_{B}(t)/(\Delta t\times \eta ch)$$ (2)

$$P_{disch}(t)=\Delta E_{B}(t)/(\Delta t\times \eta disch)$$ (3)

Where *E*_*B*_ is the battery energy, Δ*E*_*B*_ is the change in the battery energy. it is assumed that the charging efficiency (ηch) and the discharging efficiency (ηdisch) are the same. The charging power *P*_*ch*_(*t*) is usually positive while the discharging power *P*_*disch*_(*t*) is usually negative. Δ*t* represents the sampling time interval. In this research the ESSs efficiency is assumed to be 85%.

The optimal energy storage system size is shown as:

$$ESSsize=\frac{E_{Bmax}-E_{Bmin}}{DoD_{max}}$$ (4)

where *DoD*_*max*_ is the maximum depth of discharge assumed to be 80%, while *E*_*Bmax*_ and *E*_*Bmin*_ are the maximum value and the minimum value of energy respectively.

## 3. Main concepts of dandelion optimizer (do)

**Fig 3:**
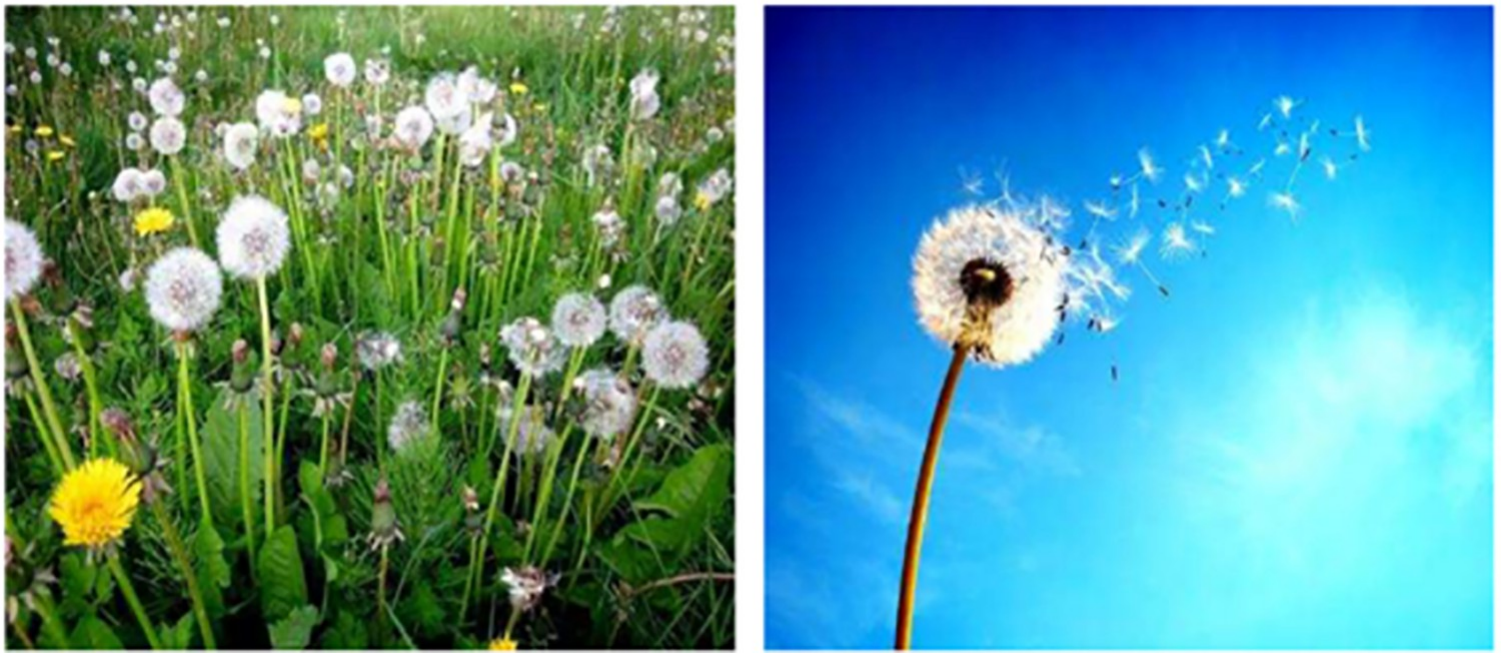
Dandelions in nature (a) growing (b) floating in the wind.

The dandelion optimizer (DO), a strategy inspired by natural processes, was introduced by Shijie Zhao in 2022 [48]. The dandelion, classified as a perennial herb in the Asteraceae family, is scientifically referred to as Herba taraxaci (see to Fig 3). The plants in question have a height that exceeds 20 centimeters at their greatest growth stage. The structures of dandelion heads have a resemblance to inflorescences. The seeds often comprise an achene, a beak, and many crested hairs. The dispersal of seeds by wind facilitates the establishment of new organisms upon reaching their complete developmental stage.

The three phases that dandelion seeds go through are listed below:

1. A vortex forms above the dandelion seed during the rising stage, and it rises while being propelled higher by wind and sunlight. There are no eddies above the seeds on a rainy day. In this instance, local searches are the only options.
2. After reaching a particular height, seeds begin to gradually drop during the descending stage.
3. Dandelions ultimately descend to the earth at random during the landing stage, where they will sprout into new plants in response to the weather and wind.

Dandelions employ a series of stages to facilitate the evolution of their population, mostly through the dispersal of their seeds to subsequent generations.

Step 1: Initialization

In the DO algorithm, each dandelion seed represents a potential solution. A DO’s population based on the dimension (dim) of the problem to be solved and the number of the generated population considering the population size and the variable’s dimension. Each potential solution between the upper bound (UB) and lower bound (LB) of the given issue is generated at random, considering dim of the problem [48].

According to the DO algorithm, the first elite member is defined as the person with the greatest fitness value, representing the optimal setting for the growth of a dandelion seed.

Step 2: Rising step

In order for seeds to float away from their parent plants during the rising stage, they must reach a certain height. Depending on factors like wind speed, air humidity, etc., dandelion seeds can grow to different heights. In this case, there are two meteorological conditions, which are as follows.

Case1:

Wind speeds may be assumed as having a lognormal distribution on a clear day. A dandelion seed’s height is influenced by the speed of the wind. The dandelion flies higher and its seeds disperse further when the wind is stronger.

$$X_{t+1}=X_{t}+\propto \times \frac{1}{e^{Ѳ}}\cos\;Ѳ\times \frac{1}{e^{Ѳ}}\sin\;Ѳ\times \ln\;Y\times (X_{s}-X_{t})$$ (5)

where *X*_*s*_ displays the randomly chosen position at iteration t upon upper and lower boundaries UB and LB, *X*_*t*_ displays the location of the dandelion seed at iteration t and Ѳ is a random number varies in the range of [-*π*, *π*]. The expression for the position that was created at random is shown as:

$$X_{s}=rand(1,\dim)\times (UB-LB)+LB$$ (6)

lnY shows a lognormal distribution subject to mean value μ = 0 and variance σ^2^ = 1.

lnY={1y2πexp[−12σ2(lny)^2]y≥00y<0 (7)

where y shows the standard normal distribution (0, 1).


$$\alpha =rand()\times (\frac{1}{T^{2}}t^{2}-\frac{2}{T}t+1)$$ (8)

The coefficients of the dandelion’s lift component are shown by *v*_*x*_ and *v*_*y*_, whereas α represents a random perturbation between [0, 1].

Case2:

Humidity and resistance of air make dandelions difficult to pull appropriately in the breeze on a wet day.


$$X_{t+1}=X_{t}\times (1-rand()*q)$$ (9)

The domain (q) can be obtained as:

$$q=\frac{1}{T^{2}-2T+1}t^{2}-\frac{1}{T^{2}-2T+1}t+1+\frac{1}{T^{2}-2T+1}$$ (10)

Finally, the mathematical formula for the ascending stage of the dandelion seed is:

$$X_{t+1}=\left\{ \begin{array}{l} X_{t}+\propto \times v_{x}\times v_{y}\times \ln\;Y\times (X_{s}-X_{t})\;randn<1.5\\ \;X_{t}\times k\;else \end{array}\right.$$ (11)

The normal distribution is followed by the random number produced by the function randn ().

Step 3: Descending step

Dandelion seeds growth to a certain height during this stage, after which they begin to slowly drop (exploration phase). In DO, Brownian motion is used to mimic the trajectory of a moving dandelion.

$$X_{t+1}=X_{t}-\alpha \times \beta_{t}\times (\frac{1}{pop}\sum_{i=1}^{pop}X_{i}-\propto \times \beta_{t}\times X_{t})$$ (12)

where *β*_*t*_ is the Brownian motion, pop is the population size.

Step 4: Landing step

In this final phase, the Dandelion algorithm centers on exploitation. Established on the outcomes of the previous two steps, the dandelion seed chooses a random spot for its landing. The more iterations there are, the closer the algorithm should go to the ideal outcome.

Finally, population evolution yields the following globally ideal response:

$$X_{t+1}=X_{elite}+s\times \frac{w\times \sigma }{{\left|t\right|}^{\left(\frac{1}{\beta }\right)}}\times \propto \times (X_{elite}-X_{t}\times \delta )$$ (13)

where *X*_*elite*_ represents the seed’s optimal position. S has a fixed constant of 0.01 whereas b is a random number with values ranging from 0 to 2, t and w are arbitrary numbers between [0, 1]. S is mathematically represented as:

$$\sigma =\frac{\Gamma (1+\beta )\times sin(\frac{\pi \beta }{2})}{\Gamma (\frac{1+\beta }{2})\times sin(\frac{\beta -1}{2})}$$ (14)

The value of *β* is 1.5, and *δ* can be found as:

$$\delta =\frac{2t}{T}$$ (15)

**Fig 4:**
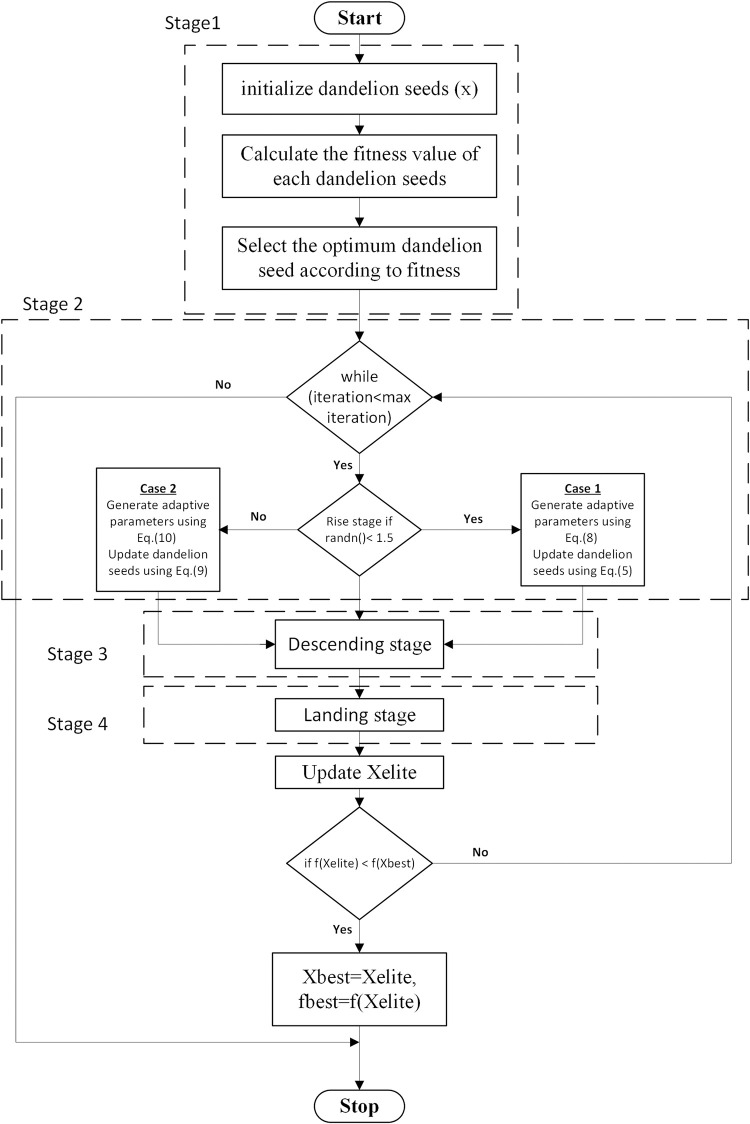
Dandelion optimizer flow chart.

Fig 4 provides the dandelion optimizer algorithm’s flow chart.

## 4. The proposed optimization problem

### 4. 1. Objective function

With today’s remarkable technological advancements, the power systems must be operated optimally. Energy storage devices are becoming increasingly important as the use of renewable sources of energy in power grids grows.

The size and location of ESSs are the variables needed to be optimal in a multi-objective optimization problem consisting of many cost objectives [49]. The objective of this optimization issue is to minimize the total cost of the system comprising Power Losses Cost (PLC), Voltage Variation Cost (*VVC*), and Peak Demand Cost (PDC) [50].


$$F=\left(\sum_{K=1}^{Nl}G_{K}[V_{i}^{2}+V_{j}^{2}-2|V_{i}||V_{j}|cos(\delta_{i}-\delta_{j})]\times C_{loss}\right)+\left(\sum_{i=1}^{Nl}(1-V_{i})\times C_{Dev}\right)+(P_{max}\times \Delta t\times C_{peak})$$ (16)

Where ***Nl*** is the maximum number of power lines that can be connected in the distribution system, ***G***_***K***_ is the transmission line k conductance value. The sending end and the receiving end line voltages are represented as ***V***_***i***_ and ***V***_***j***_, the voltage angles of the lines can be expressed as *δ*_*i*_ and *δ*_*j*_. ***P***_***max***_ is the peak value of the actual power applied to the reference bus for a specific period.

The costs associated with line losses, voltage deviation and peak demand are represented by the variables ***C***_***loss***_, ***C***_***Dev***_ and ***C***_***peak***_ respectively. The cost rates are assumed to be ***C***_***loss***_ = 284 $/*MWh*, ***C***_***Dev***_ = 142 $/*MWh* and $C_{peak}=\frac{200000\$}{MWh}/year$ [47].

### 4.2. The proposed constraints

The optimization method takes into account only the location (buses) of the energy storage units and their capacity as optimization variables. The constraints outlined in this problem can be categorized into four distinct groups: load flow restrictions, bus voltage restrictions, transmission line restrictions, and (ESS) restrictions.

The following subsections provide a comprehensive description of each of these elements.


$$P_{ch}^{t}\geq P_{Bmin}$$ (17)

$$P_{disch}^{t}\leq P_{Bmax}$$ (18)

$$E_{Bmin}\leq E_{B}^{t}\leq E_{Bmax}$$ (19)

#### 4.2.1. Energy storage constraints

The energy storage limitations outlined in Eqs (17) and (18) are designed to make sure that the charging power $P_{ch}^{t}$ does not drop below the smallest energy storage capacity ***P***_***Bmin***_, and the discharging power $P_{disch}^{t}$ does not beyond the energy storage system’s maximum power capacity ***P***_***Bmax***_. The range of values for the minimum and maximum capacities should correspond to the energy storage capacity, as indicated in Eq (19).


$$0.95\;PU\leq V_{i}^{t}\leq 1.05\;PU$$ (20)

#### 4.2.2. Bus voltage constraints

Each bus voltage over time should be between 95% and 105% of what it is rated for.


$$S_{l}^{t}\leq S_{lmax}$$ (21)

#### 4.2.3. Transmission lines constraints

The maximum loading for each line ***S***_***lmax***_ should not be exceeded over time.


$$P_{Gmin}\leq P_{G}^{t}\leq P_{Gmax}$$ (22)

$$Q_{Gmin}\leq Q_{G}^{t}\leq Q_{Gmax}$$ (23)

#### 4.2.4. Power constraints

According to the capacity limitations, the reactive and active powers of each power plant should fluctuate between their permitted maximum and minimum limits.

## 5. Simulation results and analysis

**Fig 5:**
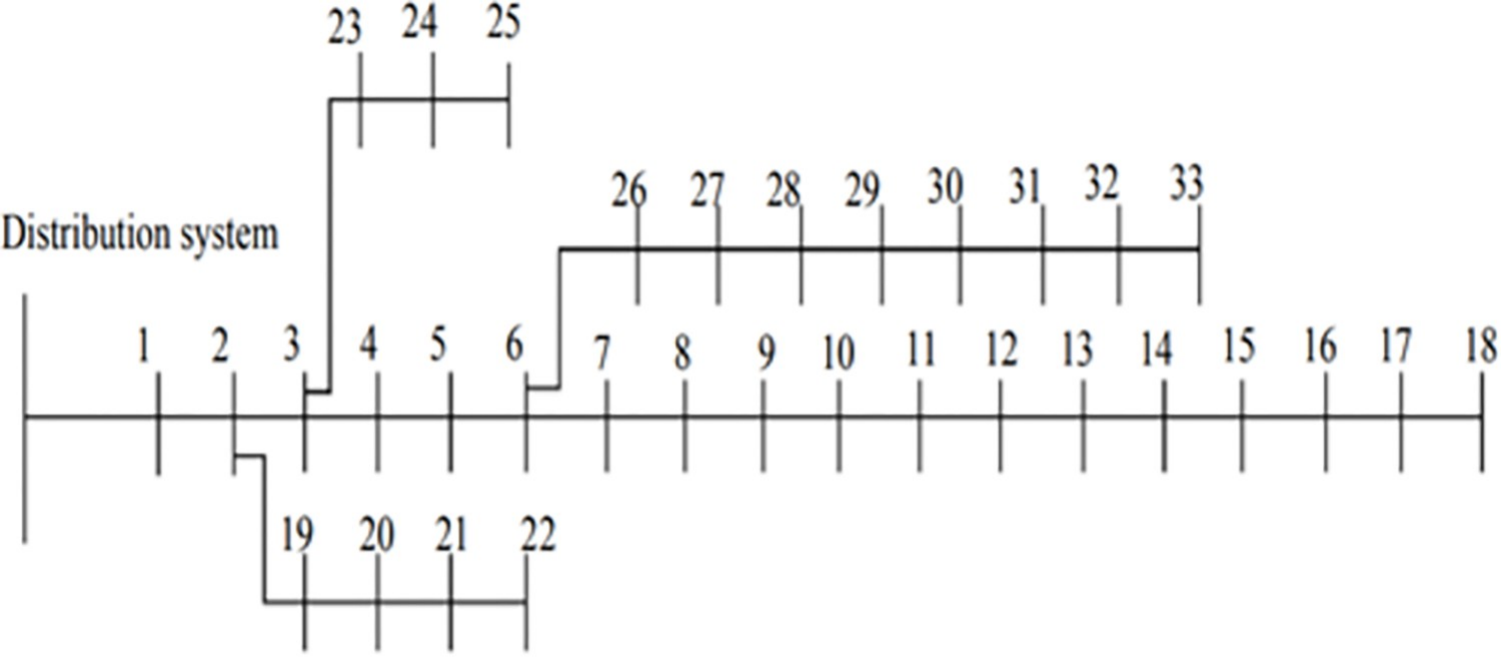
Test system for IEEE 33 bus distribution.

The effects of the proposed ESS deployment on system voltage profile, system performance, and cost containment are examined in this section. Three different case studies are examined for the performance analysis: Case 1 is the basic scenario (without ESSs), scenario 2 is the placement of ESSs using ALO optimization algorithm, and Case 3 is the placement of ESSs using the proposed DO optimizer. In this simulation, the IEEE 33 bus test system is utilized (S1 Table) [51]. The bus system’s organizational structure includes 33 bus radials, 32 lines, 1 slack bus at 12.66 kV base voltage and 100 MVA base power. 3.71 MW is the total real power, and 2.31 MVAr is the reactive power. An IEEE 33 bus is depicted in a single line diagram in Fig 5.

**Fig 6:**
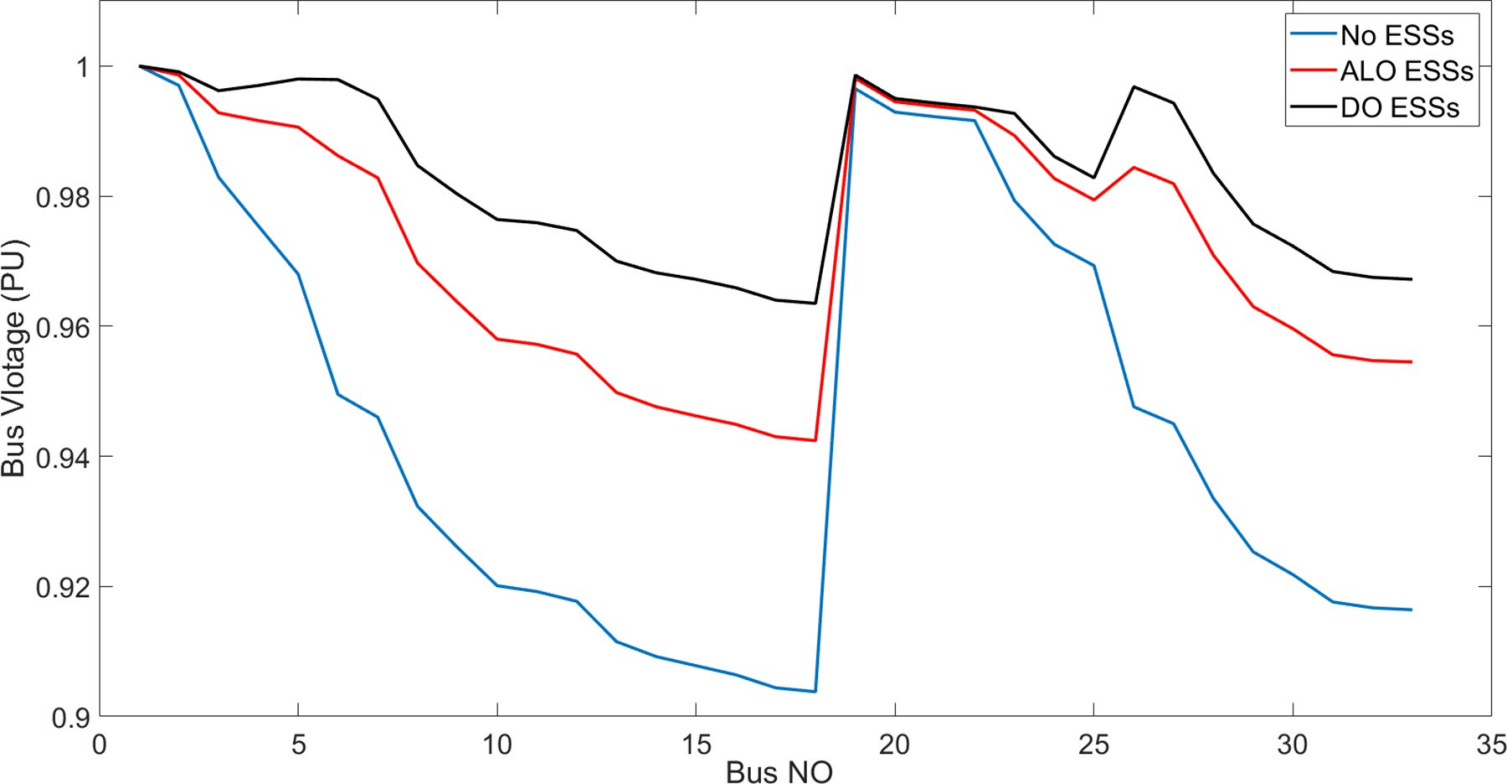
IEEE 33 Bus voltage profile a) with no ESSs b) with ALO ESSs optimizer c) with proposed DO ESSs optimizer.

**Fig 7:**
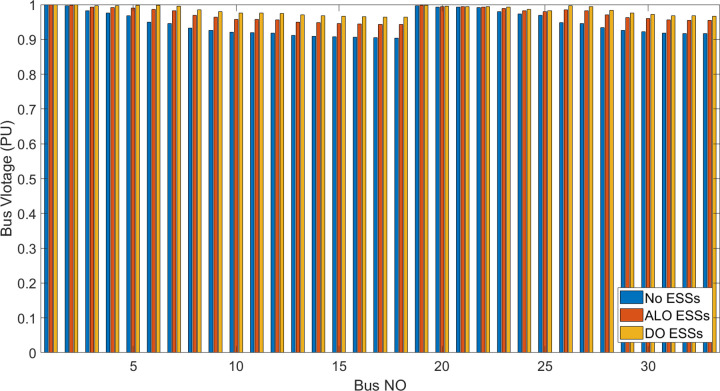
Dissimilarity of voltage profile for IEEE 33 bus with and without ESSs using ALO and DO.

**Fig 8:**
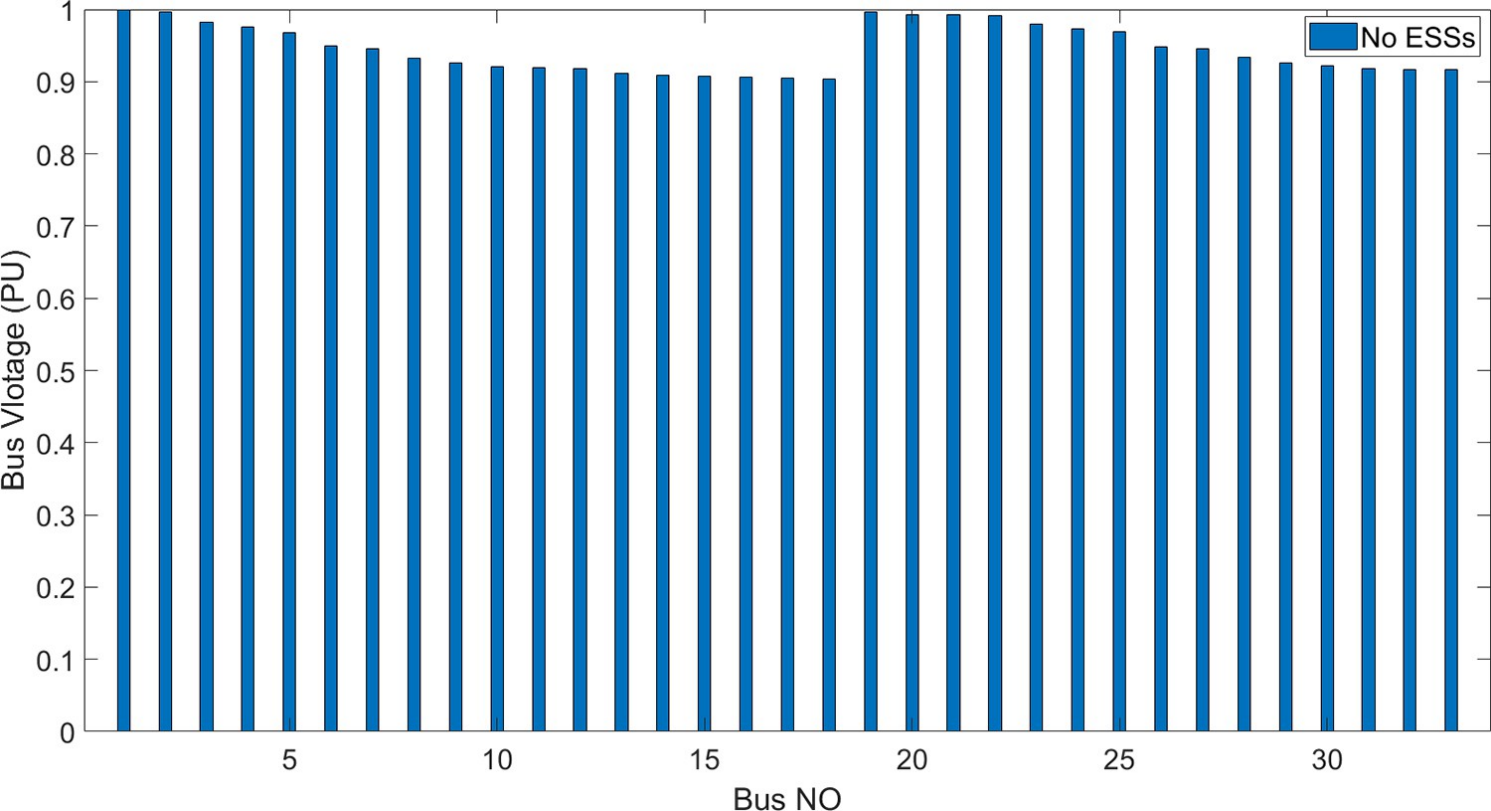
Deviation of voltage profile for IEEE 33 bus without ESSs.

**Fig 9:**
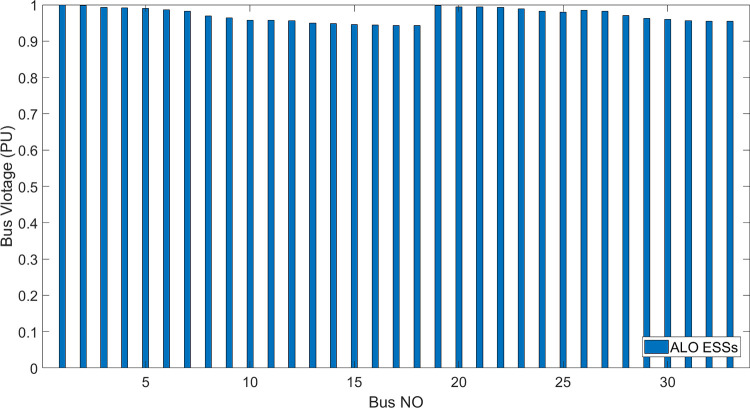
Deviation of voltage profile for IEEE 33 bus with ESSs using ALO algorithm.

**Fig 10:**
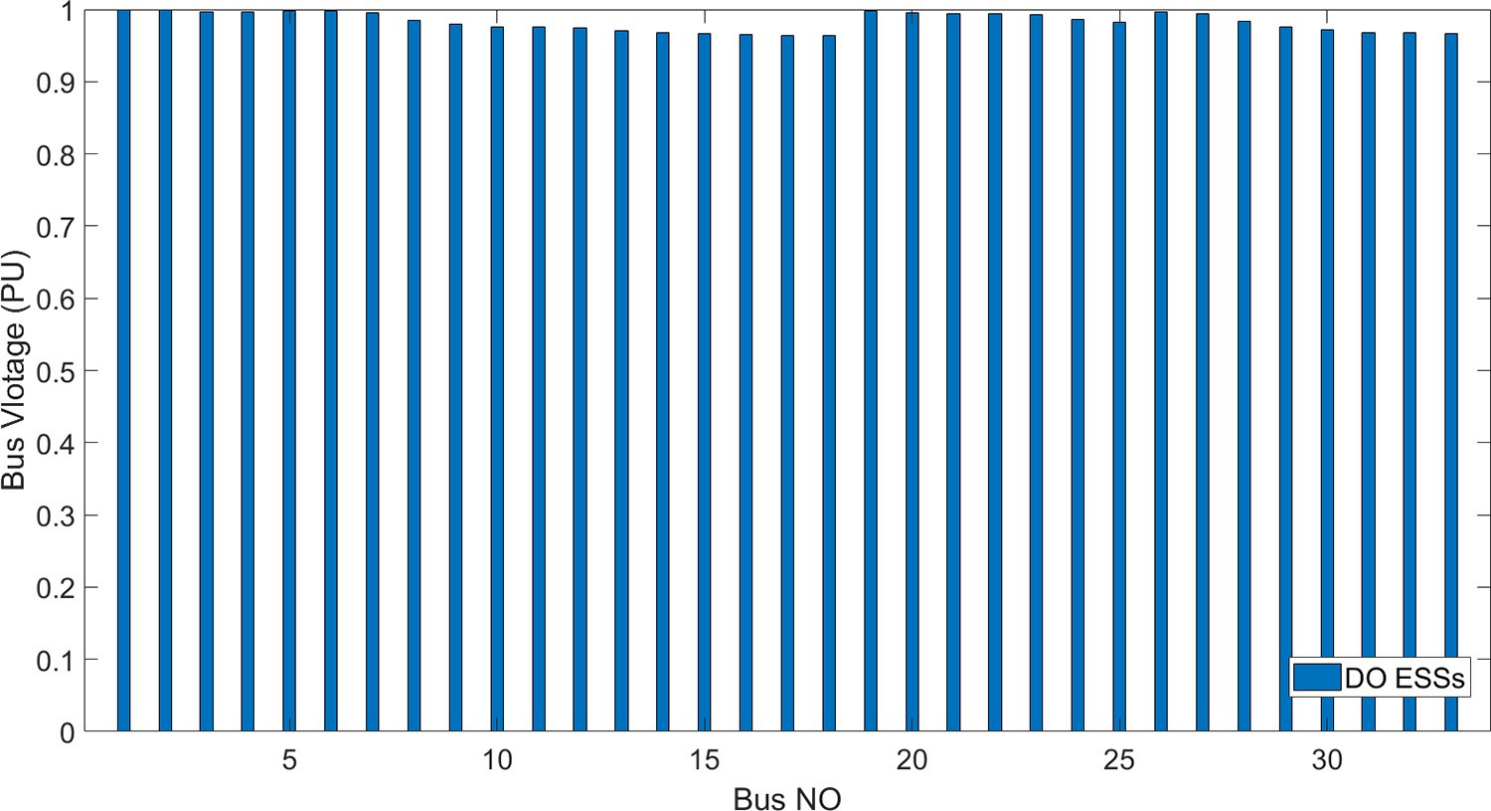
Deviation of voltage profile for IEEE 33 bus with ESSs using DO algorithm.

Fig 6 shows the bus voltage for three cases: first, without installing ESSs; second, after installing the ESSs via ALO optimization algorithm and third, the voltage profile acquired by the implemented (DO) technique. The analysis of Fig 6 reveals that the installation of (ESS) has a positive effect on the voltage profile of the network. Fig 7 illustrates the voltage change in the buses resulting from the three circumstances, whereas Figs 8–10 depict the individual bus voltages for each respective case.

The effectiveness of the suggested methodology is assessed by comparing the system outcomes with those obtained from an alternative strategy, such as the ALO method. Table 1 displays a comprehensive examination of the results acquired from the original system and the later integration of ESSs employing ALO and the suggested methodology for assessing power loss, minimum voltage, and operational expenses of the system. The results show that the DO achieves net saving of 1.7% in total cost than ALO with better voltage profile.

**Table 1:** Optimum size and location of ESSs for IEEE 33 bus system.

Algorithms	Bus Location	ESS size (MWh)	Minimum voltage (pu)	Total Operating Cost $/year	Power Loss (KW)
Without DG	---	----	0.9038	----	211
ALO	26	2.4507	0.9424	50167.5	111.0320
DO	6	2.5902	0.9635	49320.1	111.0320

**Table 2** illustrates the impact of altering the number of ESS on the 33-bus system’s optimal solution.

**Table 2:** Size and placement of ESSs optimal for IEEE 33 bus systems.

The number of ESSs that can be provided.	ALO based optimal allocation of ESSs		Total Operating Cost of ESSs $/year	Elapsed time by algorithm to find optimal solution in seconds	The number of ESSs that can be provided.	DO based optimal allocation of ESSs		Total Operating Cost of ESSs $/year	Elapsed time by algorithm to find optimal solution in seconds
	Bus no.	Size MWh				Bus no.	Size MWh		
1	26	2.4507	50167.5	23.818414	1	6	2.5902	49320.1	16.695445
5	6	2.59023	49320.1	24.711285	5	6	2.59023	49320.1	17.278905
	5	3.63091				4	0.100124		
	14	0.820672				11	0.135889		
	7	2.34196				15	0.10601		
	33	0.948381				26	0.566627		
10	6	2.59023	49.3201	28.188763	10	6	2.59023	49320.1	17.327187
	32	2.87756				26	0.166591		
	18	2.6756				33	0.101612		
	24	1.77831				22	0.1		
	14	0.70665				1	1.96496		
	25	3.15176				10	0.923314		
	16	1.18055				24	1.29678		
	22	1.86802				32	2.39981		
	7	2.25449				29	1.70646		
	10	2.01558				19	0.346829		

## 6. Conclusion

The installation of ESSs into a distribution network has the potential to dramatically improve the network’s overall energy efficiency. It is possible to increase the overall performance of the network by intelligently sizing and installing these ESSs in the proper areas. This will be possible if you follow the steps outlined in this paper. The capacity of these ESSs to meet peak energy demand, make use of renewable energy sources, ensure power quality, and save costs associated with the installation of distribution networks is contingent on their being adequately sized and strategically positioned. The Dandelion Optimizer (DO) is utilized in the suggested method that is presented in this article in order to realize this objective as its ultimate destination. An IEEE 33 bus distribution system was utilized in the evaluation of the method, and the findings demonstrated that its performance was superior to that of the initial system achieving lower cost, better voltage profile and even superior to that of the Ant Lion Optimizer (ALO). Because of how easy it is to implement this technique and how well it handles the difficulties involved with optimization, the selected ESS sites and sizes have the potential to be used in the real deployment of the distribution networks.

## Supporting information

S1 Table IEEE 33 bus distribution test system bus data [51]. (DOCX)
