# Supplementary material for: Optimal allocation of distributed energy storage systems to enhance voltage stability and minimize total cost
Source: PLoS One. 2024 Jan 29;19(1):e0296988. doi: 10.1371/journal.pone.0296988 (PMC10824428; doi:10.1371/journal.pone.0296988)
Supplement: S1 Table — (DOCX) [file pone.0296988.s001.docx]

**TABLE S1: IEEE 33 Bus Distribution Test System Bus Data.**

| Bus Number |  | Type | Maximum Voltage | Minimum Voltage | Active Demand | Reactive Demand |
| --- | --- | --- | --- | --- | --- | --- |
| 1 |  | reference | 1 | 1 | 0 | 0 |
| 2 |  | PQ | 1.05 | 0.95 | 0.1 | 0.06 |
| 3 |  | PQ | 1.05 | 0.95 | 0.09 | 0.04 |
| 4 |  | PQ | 1.05 | 0.95 | 0.12 | 0.08 |
| 5 |  | PQ | 1.05 | 0.95 | 0.06 | 0.03 |
| 6 |  | PQ | 1.05 | 0.95 | 0.06 | 0.02 |
| 7 |  | PQ | 1.05 | 0.95 | 0.2 | 0.1 |
| 8 |  | PQ | 1.05 | 0.95 | 0.2 | 0.1 |
| 9 |  | PQ | 1.05 | 0.95 | 0.06 | 0.02 |
| 10 |  | PQ | 1.05 | 0.95 | 0.06 | 0.02 |
| 11 |  | PQ | 1.05 | 0.95 | 0.045 | 0.03 |
| 12 |  | PQ | 1.05 | 0.95 | 0.06 | 0.035 |
| 13 |  | PQ | 1.05 | 0.95 | 0.06 | 0.035 |
| 14 |  | PQ | 1.05 | 0.95 | 0.12 | 0.08 |
| 15 |  | PQ | 1.05 | 0.95 | 0.06 | 0.01 |
| 16 |  | PQ | 1.05 | 0.95 | 0.06 | 0.02 |
| 17 |  | PQ | 1.05 | 0.95 | 0.06 | 0.02 |
| 18 |  | PQ | 1.05 | 0.95 | 0.09 | 0.04 |
| 19 |  | PQ | 1.05 | 0.95 | 0.09 | 0.04 |
| 20 |  | PQ | 1.05 | 0.95 | 0.09 | 0.04 |
| 21 |  | PQ | 1.05 | 0.95 | 0.09 | 0.04 |
| 22 |  | PQ | 1.05 | 0.95 | 0.09 | 0.04 |
| 23 |  | PQ | 1.05 | 0.95 | 0.09 | 0.05 |
| 24 |  | PQ | 1.05 | 0.95 | 0.42 | 0.2 |
| 25 |  | PQ | 1.05 | 0.95 | 0.42 | 0.2 |
| 26 |  | PQ | 1.05 | 0.95 | 0.06 | 0.025 |
| 27 |  | PQ | 1.05 | 0.95 | 0.06 | 0.025 |
| 28 |  | PQ | 1.05 | 0.95 | 0.06 | 0.02 |
| 29 |  | PQ | 1.05 | 0.95 | 0.12 | 0.07 |
| 30 |  | PQ | 1.05 | 0.95 | 0.2 | 0.6 |
| 31 |  | PQ | 1.05 | 0.95 | 0.15 | 0.07 |
| 32 |  | PQ | 1.05 | 0.95 | 0.21 | 0.1 |
| 33 |  | PQ | 1.05 | 0.95 | 0.06 | 0.04 |

**TABLE 2: Branches Data of the IEEE 33 Bus Distribution Test System.**

| Branch No | From | To | R (pu) | X(pu) |
| --- | --- | --- | --- | --- |
| 1 | 1 | 2 | 0.0575 | 0.0293 |
| 2 | 2 | 3 | 0.3076 | 0.1566 |
| 3 | 3 | 4 | 0.2284 | 0.1163 |
| 4 | 4 | 5 | 0.2378 | 0.1211 |
| 5 | 5 | 6 | 0.5110 | 0.4411 |
| 6 | 6 | 7 | 0.1168 | 0.3861 |
| 7 | 7 | 8 | 1.0680 | 0.7710 |
| 8 | 8 | 9 | 0.6426 | 0.4617 |
| 9 | 9 | 10 | 0.6514 | 0.4617 |
| 10 | 10 | 11 | 0.1227 | 0.0406 |
| 11 | 11 | 12 | 0.2336 | 0.0772 |
| 12 | 12 | 13 | 0.9159 | 0.7206 |
| 13 | 13 | 14 | 0.3379 | 0.4448 |
| 14 | 14 | 15 | 0.3687 | 0.3282 |
| 15 | 15 | 16 | 0.4656 | 0.3400 |
| 16 | 16 | 17 | 0.8042 | 1.0738 |
| 17 | 17 | 18 | 0.4567 | 0.3581 |
| 18 | 18 | 19 | 0.1023 | 0.0976 |
| 19 | 19 | 20 | 0.9385 | 0.8457 |
| 20 | 20 | 21 | 0.2555 | 0.2985 |
| 21 | 21 | 22 | 0.4423 | 0.5848 |
| 22 | 22 | 23 | 0.2815 | 0.1924 |
| 23 | 23 | 24 | 0.5603 | 0.4424 |
| 24 | 24 | 25 | 0.5590 | 0.4374 |
| 25 | 25 | 26 | 0.1267 | 0.0645 |
| 26 | 26 | 27 | 0.1773 | 0.0903 |
| 27 | 27 | 28 | 0.6607 | 0.5826 |
| 28 | 28 | 29 | 0.5018 | 0.4371 |
| 29 | 29 | 30 | 0.3166 | 0.1613 |
| 30 | 30 | 31 | 0.6080 | 0.6008 |
| 31 | 31 | 32 | 0.1937 | 0.2258 |
| 32 | 32 | 33 | 0.2128 | 0.3308 |
